# Have giant lobelias evolved several times independently? Life form shifts and historical biogeography of the cosmopolitan and highly diverse subfamily Lobelioideae (Campanulaceae)

**DOI:** 10.1186/1741-7007-7-82

**Published:** 2009-11-26

**Authors:** Alexandre Antonelli

**Affiliations:** 1 Department of Plant and Environmental Sciences, University of Gothenburg, Box 461, 40530, Göteborg, Sweden; 2 Present address : University of Zurich, Institute of Systematic Botany, Zollikerstrasse 107, CH 8008, Zürich, Switzerland

## Abstract

**Background:**

The tendency of animals and plants to independently develop similar features under similar evolutionary pressures - convergence - is a widespread phenomenon in nature. In plants, convergence has been suggested to explain the striking similarity in life form between the giant lobelioids (Campanulaceae, the bellflower family) of Africa and the Hawaiian Islands. Under this assumption these plants would have developed the giant habit from herbaceous ancestors independently, in much the same way as has been suggested for the giant senecios of Africa and the silversword alliance of Hawaii.

**Results:**

Phylogenetic analyses based on plastid (*rbc*L, *trn*L-F) and nuclear (internal transcribed spacer [ITS]) DNA sequences for 101 species in subfamily Lobelioideae demonstrate that the large lobelioids from eastern Africa the Hawaiian Islands, and also South America, French Polynesia and southeast Asia, form a strongly supported monophyletic group. Ancestral state reconstructions of life form and distribution, taking into account phylogenetic uncertainty, indicate their descent from a woody ancestor that was probably confined to Africa. Molecular dating analyses using Penalized Likelihood and Bayesian relaxed clock approaches, and combining multiple calibration points, estimate their first diversification at ~25-33 million years ago (Ma), shortly followed by several long-distance dispersal events that resulted in the current pantropical distribution.

**Conclusion:**

These results confidently show that lobelioid species, commonly called 'giant', are very closely related and have not developed their giant form from herbaceous ancestors independently. This study, which includes the hitherto largest taxon sampling for subfamily Lobelioideae, highlights the need for a broad phylogenetic framework for testing assumptions about morphological development in general, and convergent evolution in particular.

## Background

When the great 18th-century naturalist Carl Linnaeus took a closer look at a the whale during the preparation of his natural classification system, he realized for the first time that it was not a fish but a mammal [1]. Its form and fins were certainly like the fish, but it also had mammary glands and lungs and should, thus, be classified as a mammal. Ever since Linnaeus, what we today refer to as convergent evolution (see, for example, [2,3]) has been advocated as a common and widespread phenomenon in nature. From single features (such as the wings of birds and insects or the cones of cycads and conifers) to whole organisms (shrimps and krill, cacti and euphorbias), superficial similarity has been repeatedly demonstrated to evolve independently in distantly related evolutionary lineages.

**Figure 1:**
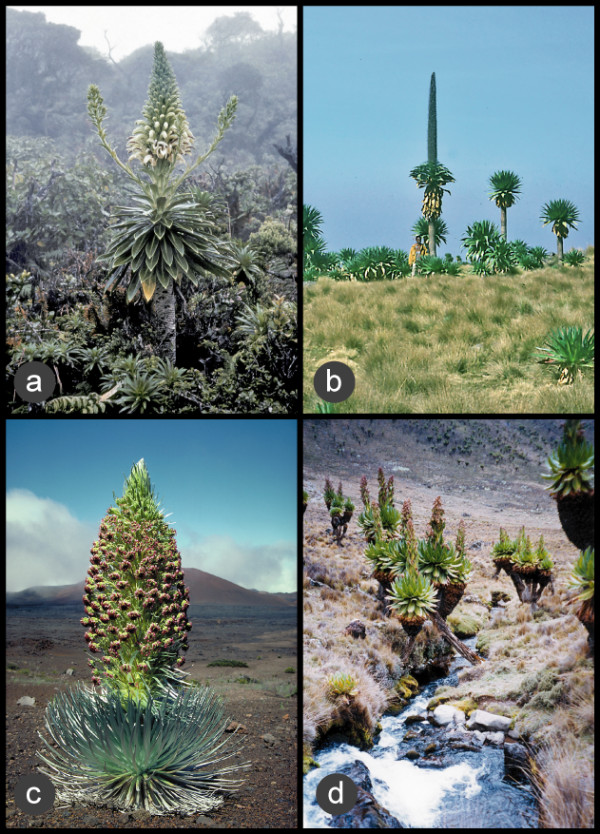
**Same life form, same history? **Giant lobelioids (Campanulaceae: Lobelioideae) from the Hawaiian Islands (a) have been suggested to have converged into the giant life form independently from the giant lobelias of Africa (b), in much the same way as the silversword alliance of Hawaii (c) and the giant senecios of Africa (d) in family Asteraceae. [Credits: a, *Lobelia gloria-montis *by Frederick R. Warshauer; b, *Lobelia rhynchopetalum *by Christian Puff; c, *Argyroxiphium sandwicense *by Gerald D. Carr; d, *Dendrosenecio keniodendron*, from http://www.wikipedia.org].

In plants, the giant lobelias from the Hawaiian Islands and tropical Africa have been cited as remarkable examples of morphological convergence in the family Campanulaceae (the bellflower family). According to some earlier authors [4-6], convergence from herbaceous plants into tall treelets would have occurred independently in different mountain systems in response to similar tropical alpine climates consisting of nightly frosts and rapid temperature fluctuations. The idea that many morphological features in giant lobelias represent adaptations to proximal environmental factors [7] is based on the observation that the leaf rosettes of many giant species provide insulation for the central axis, protecting the shoot apex and the young leaves and inflorescences from extreme temperatures. According to Hedberg [4], a similar evolutionary pattern can be observed between the giant senecios of Africa and the silversword alliance of the Hawaiian Islands (both in Asteraceae), which Hedberg cited as an example of convergent evolution alongside the giant lobelias (Figure 1).

Since these pioneering works, many studies have addressed specific aspects of giant lobelioids, not only providing novel insights into the conspicuous habit of the giant forms [6] but also their detailed morphology [8-10], chromosome numbers [11,12], conservation status [13] and physiology [14-17]. Based on a phylogenetic analysis of 17 species in subfamily Lobelioideae (including 13 giant forms and four herbaceous taxa), Knox *et al*. [18] proposed that the giant lobelioids consisted of a Chilean hexaploid group and a pantropical tetraploid group, all derived from herbaceous ancestors. Later, using the Chilean giant species as outgroup and analysing the relationships and biogeographic history among 21 African and one Brazilian giant lobelia, Knox and Palmer [7] suggested that the eastern African giant lobelias arrived in Africa from the Asian/Pacific region. More recently, Givnish *et al*. [10] used a larger data set (including 38 lobelioid species, of which two are herbaceous) to demonstrate that all Hawaiian lobelioids constitute a monophyletic clade, thus corroborating previous results by Givnish and co-workers [8,19]

Despite these major advances, we still need to verify whether the biogeographic and phylogenetic patterns obtained so far for Lobelioideae will stand the inclusion of increased taxon sampling. In order to confidently test whether the giant forms worldwide evolved independently from herbaceous ancestors, it seems vital to include a considerably larger representation of herbaceous taxa and to root the Lobelioideae tree with representative taxa from outside the subfamily.

Here I build on previous studies to reconstruct the life form and biogeographic evolution of Lobelioideae. I focus on the following questions: (i) have giant lobelioids evolved from herbaceous ancestors several times independently?; (ii) when and where did the giant habit evolve?; and (iii) is the life form and geographic distribution phylogenetically conservative? To address these questions, I generated fast-evolving nuclear sequence data for several giant species and, in combination with all available DNA sequences for selected markers, I performed phylogenetic, biogeographic, character evolution and molecular dating analyses. The results reported here demonstrate that the giant lobelioids have a very different evolutionary history than the giant Asteraceae.

## Results and discussion

### Phylogeny

**Figure 2:**
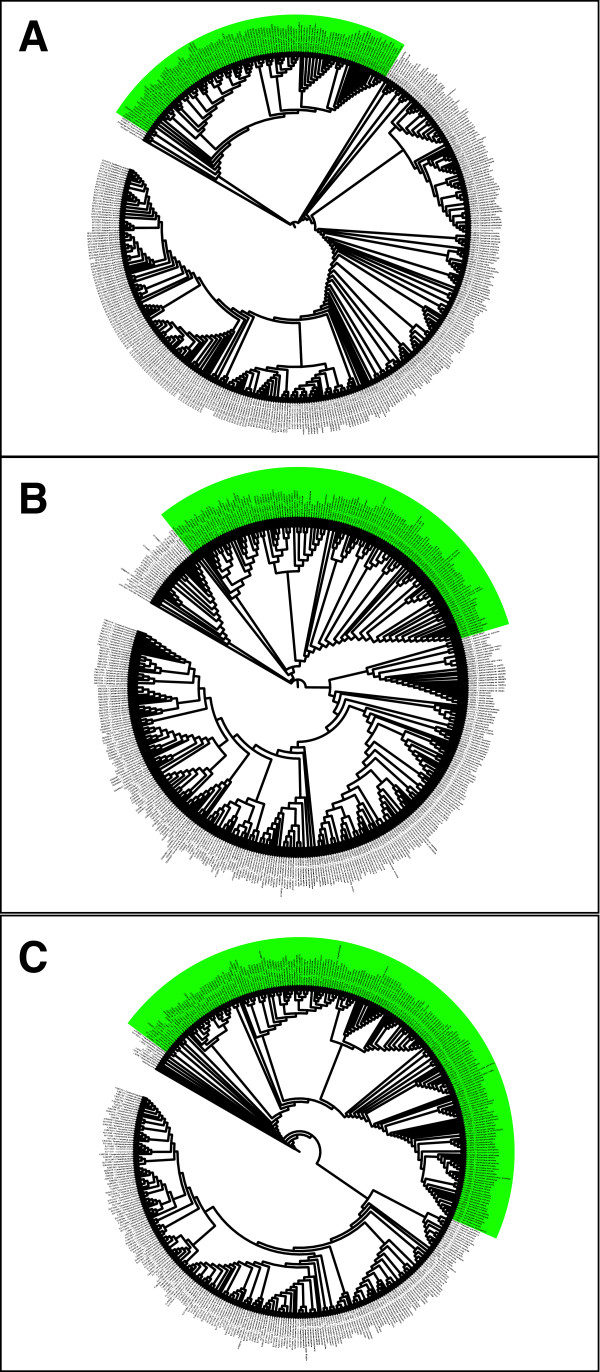
**Maximum Likelihood trees of the Campanulaceae**. Cladograms with the highest likelihood scores yielded from 10 independent runs in the software GARLI, based on: (a) *trn*L-F, 452 sequences; (b) ITS, 445 sequences; and (c) *rbc*L, 438 sequences. Subfamily Lobelioideae is highlighted in green. GenBank accession numbers are given following the species names (as stored in GenBank).

**Figure 3:**
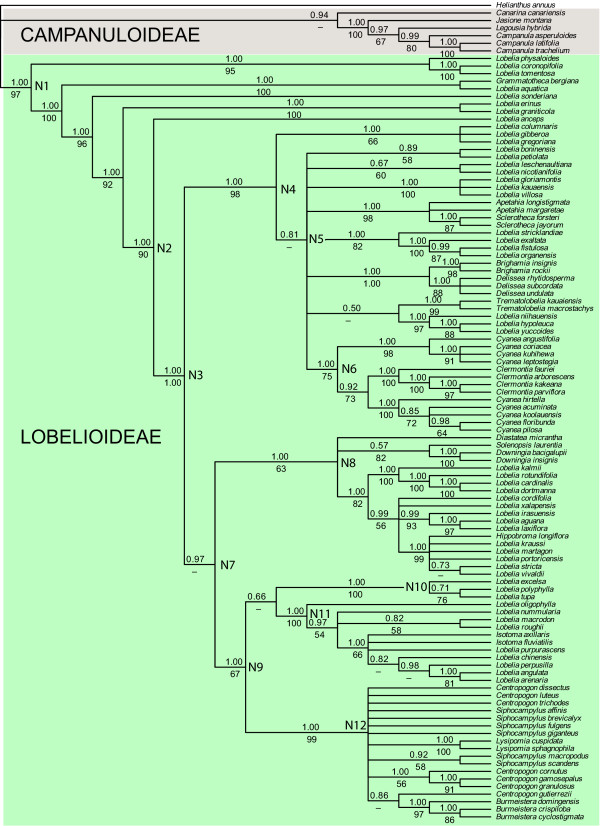
**Phylogeny of subfamily Lobelioideae**. Fifty-percent majority-rule consensus cladogram from the Bayesian analysis based on the combined data set (*trn*L-F, ITS, *rbc*L). Numbers above branches indicate Bayesian posterior probabilities; numbers below branches represent bootstrap support values (> 50) calculated under maximum parsimony. Key nodes discussed in the text are labelled N1--N12.

The maximum likelihood trees based on all Campanulaceae sequences available at GenBank and complemented by novel sequences generated in this study for *trn*L-F, ITS and *rbc*L are shown in Figure 2a, c. Lobelioideae is retrieved as monophyletic in all three analyses of each marker separately, providing an indication of the choice of taxa for the subsequent analyses. The results from the Bayesian and bootstrap analyses on the Lobelioideae data set are shown together in Figure 3. Generally, strongly supported relationships were in agreement with, or at least did not contradict, previous phylogenetic analyses [7,8,10,18-21]. The few differences obtained here (especially in clade N4, Figure 3) are discussed below.

### Molecular dating

**Figure 4:**
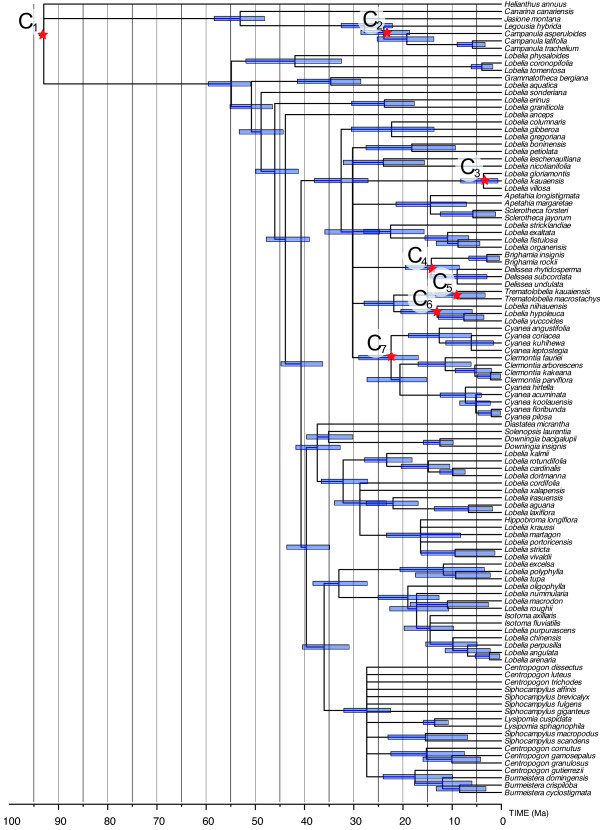
**Divergence time estimates using Penalized Likelihood**. Mean age chronogram showing 95% confidence intervals of age nodes (bars), based on 1000 Bayesian trees from a post burn-in tree sample. The stars represent calibration points: C_1_, crown age of Asterales as estimated by Bremer *et al*. [66], fixed age = 93 Ma; C_2_, crown age of subfamily Campanuloideae, based on a fossil *Campanula*, minimal age = 5.33 Ma [68]; C_3 _-- C_7_, diversification of Hawaiian taxa, corresponding to the age of the oldest island of the Hawaiian Ridge (Kure) after which a continuous chain of islands has been available as 'stepping stones' for propagules of the Hawaiian biota, maximum age = 29.8 Ma [26].

**Figure 5:**
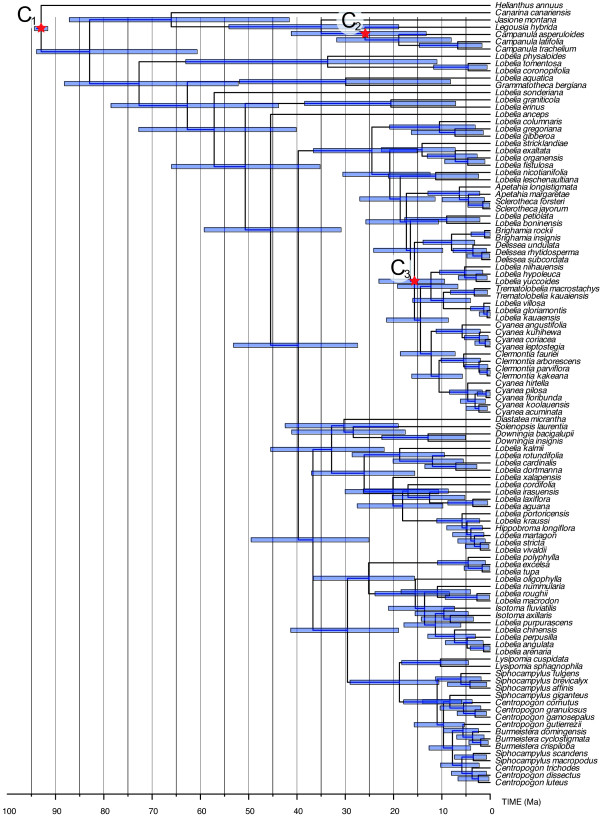
**Divergence time estimates using Bayesian relaxed clock (BEAST)**. Tree, with the maximum sum of clade credibilities and branch lengths equal to the median ages as calculated from 40,000 post burn-in chronograms. Bars show 95% Highest Posterior Density intervals of age nodes. Calibration points as in the previous figure, with the following exceptions: (i) that the tree prior incorporated for the root of the tree (C_1_, 93 Ma) was not constrained *a priori *on a particular clade, but allowed to be calculated in the phylogenetic and dating estimation; and (ii) that all Hawaiian species were constrained as monophyletic prior to the analysis, following the results by Givnish *et al*. [10] (see Methods).

**Figure 6:**
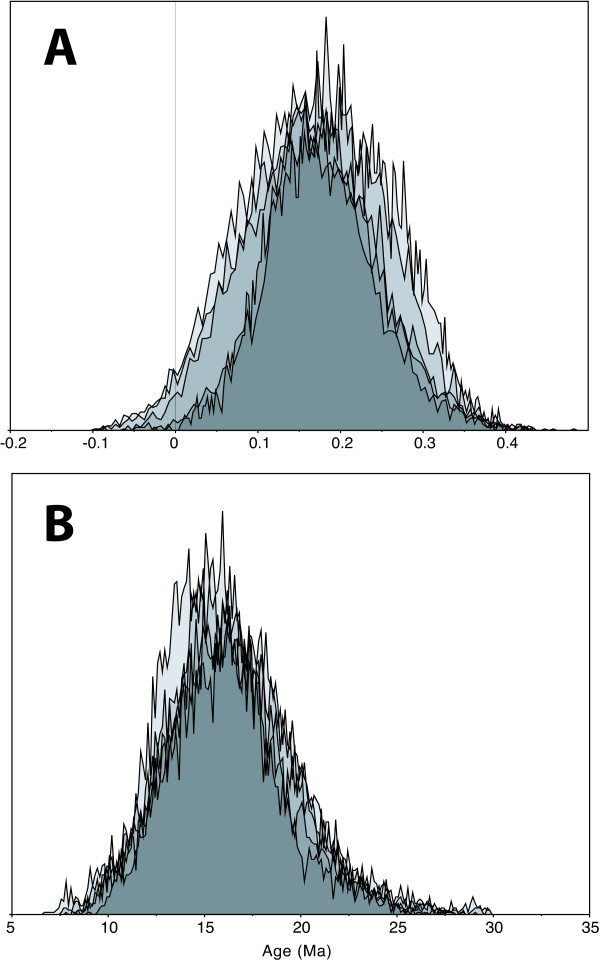
**Selected statistics from the BEAST analysis**. Distribution curves obtained from a post burn-in sample of 40,000 chronograms; each curve represents an independent run. (a) Covariance between parent and child branch rates. A value close to zero indicates that there is no support for autocorrelation (a main assumption in Penalized Likelihood analyses), meaning that the BEAST results here are probably more realistic. (b) Distribution of age estimates for the crown group of the Hawaiian taxa.

The results from the molecular dating analyses are summarized in Table 1. Generally, node age estimations exhibited relatively large confidence intervals, both under the Penalized Likelihood (Figure 4) and the Bayesian relaxed clock (Figure 5) methods. The tree with highest sum of clade credibilities generated under the Bayesian evolutionary analysis by sampling trees (BEAST) analysis (Figure 5; effective sample size = 322) did not exhibit any strongly supported phylogenetic conflict with the consensus cladogram of the MrBayes analysis (Figure 3). Although there was great variation between the age estimates obtained in Penalized Likelihood as compared to BEAST, in all key nodes listed in Table 1 there was considerable overlap of the 95% confidence intervals and highest probability densities. The mean covariance between parental and child lineages in the BEAST analysis was 0.157 (Figure 6a). Since a covariance close to zero indicates no significant autocorrelation of rates (the fundamental assumption made by Penalized Likelihood), this indicates that the BEAST results reported here are probably more realistic than the ages obtained in Penalized Likelihood [22]. For comparison, both results are reported throughout the paper.

**Table 1:** Crown group ages (in million of years) of the major groups outlined in Figure 3.

	Penalized Likelihood		BEAST	
	
Clade	Mean	95% CI	Median	95% HPD
**N1**	54.9	50.9 -- 59.6	72.7	52.2 -- 88.2

**N2**	43.9	39.0 -- 47.8	45.5	30.9 -- 59.2

**N3**	40.7	36.3 -- 44.8	39.7	27.4 -- 53.1

**N4**	32.7	27.1 -- 38.0	24.5	15.1 -- 36.6

**N5**	30.3	24.8 -- 35.9	20.8	12.4 -- 30.5

**N6**	22.4	16.9 -- 29.0	12.2	5.74 -- 16.2

**N7**	39.6	34.9 -- 43.6	36.7	25.1 -- 49.5

**N8**	37.4	32.8 -- 41.8	32.8	22.0 -- 45.4

**N9**	39.0	31.0 -- 40.4	29.6	18.9 -- 41.3

**N10**	11.8	3.48 -- 20.6	4.56	0.95 -- 10.9

**N11**	19.0	12.7 -- 25.1	15.5	8.43 -- 23.8

**N12**	27.4	22.5 -- 32.0	18.8	10.6 -- 29.0

BEAST = Bayesian evolutionary analysis by sampling trees; CI = confidence interval; HPD = highest posterior density.

**Figure 7:**
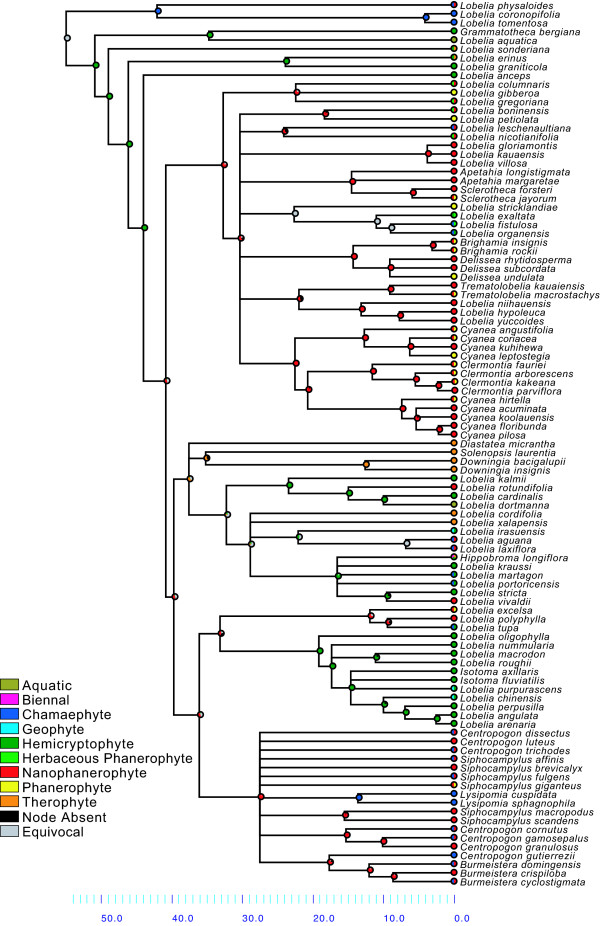
**Life form shifts in relation to time**. Results from character state reconstructions in subfamily Lobelioideae. The pie charts on each node show the relative proportion of character assignments based on the individual results from the Fitch optimization of 1000 Bayesian post burn-in trees, counting uniquely best states. States of extant species are shown before each species name. Results plotted on the mean age chronogram obtained using Penalized Likelihood (Figure 4). See Table 2 for a definition of life forms.

### Life form shifts

**Figure 8:**
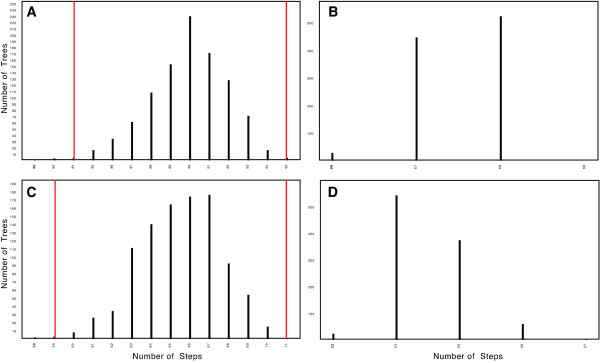
**Tests of phylogenetic conservatism in life form and geographic distribution**. Comparison of the minimal number of steps required to reconstruct life form and geographic distribution on a sample of 1000 simulated trees (generated by keeping the tree topology as in the Bayesian consensus, but randomly shuffling character states) with 1000 empirical trees (randomly chosen from the post burn-in Bayesian sample). (a) Simulated results for life form; (b) empirical results for life form; (c) simulated results for geographic distribution; and (d) empirical results for geographic distribution. For both characters, the observed values fall outside the lower percentiles of the simulated curves (indicated by the red lines in (a) and (c); *P *< 0.001), indicating that both life form and geographic distribution are phylogenetically conservative.

The character state reconstruction of life form is shown in Figure 7 (see Table 2 for the definitions). The reconstruction of life form on the Bayesian cladogram required 68 steps and the character is phylogenetically conservative (*P *< 0.001; Figure 8a, b). The ancestral life form of the most recent common ancestor (MRCA) of Lobelioideae (N1 in Figure 3) is ambiguous, due to an early split between a chamaephyte lineage (leading to the clade *Lobelia physaloides - L. tomentosa*) and a hemicryptophyte lineage (the ancestor to all the others).

**Table 2:** Characterization of life forms used in the ancestral state reconstructions (from Lammers [23]).

Life form	Stems	Buds
Aquatic	Comprises both Hydrophytes and Hydrohemicryptophytes	

Chamaephyte	Herbaceous and/or woody and persistent	On or just above soil level but never more than 0.5 m above ground

Geophyte	Hemicryptophytes that survive unfavourable seasons in the form of a rhizome, bulb, tuber or root bud	Below soil level

Hemicryptophyte	Herbaceous, often dying back after the growing season but with buds or growth at soil level	Just on or below soil level

Herbaceous phanerophyte	Herbaceous and persisting for several years	Above soil level

Hydrophyte	Vegetative shoots entirely in water, the leaves usually submersed and/or floating; flower-bearing parts may emerge above the water	Permanently or temporarily on the bottom of the water

Nanophanerophyte	Woody and indefinitely persistent	Above soil level but normally less than 3 m above ground

Phanerophyte	Woody and indefinitely persistent	Normally 3 m or more above ground

Therophyte	Plants surviving unfavourable seasons in the form of seeds, completing their life-cycle during the favourable season	

There is great life form variation in the Lobelioideae at the intergeneric, intrageneric and intraspecific levels (Figure 7, Table 3). Only in a few cases is there a homogeneous life form correlating with a certain clade, as indicated by the low Consistency Index of life form overall (0.27 on the tree shown in Figure 7). One example of local high consistency is in the clade *Lobelia oligophylla - L. arenaria *(N11 in Figure 3), in which all exhibit the hemicryptophyte habit, at least facultatively. Although the overall low consistency is partly biased by an uneven taxon sampling (for example, there are only two species of *Lysipomia *sampled but all 30 species are strictly chamaephytes), it does reflect a general pattern of difficulties in identifying natural taxa in Lobelioideae based on morphology (see, for example, [21,23]).

**Table 3:** List of species used in the analyses of the Lobelioideae data set, their life form and distribution (simplified from Lammers [23]), and GenBank accession numbers.

	Taxon	Life form	Distribution	rbcL	trnL-trnF	ITS
1	*Apetahia longistigmata*	Nanophanerophyte	French Polynesia	DQ285272	DQ285155	
2	*Apetahia margaretae*	Nanophanerophyte	French Polynesia	DQ285286	DQ285169	
3	*Brighamia insignis*	Nanophanerophyte or Phanerophyte	Hawaiian islands	AF042664	DQ356189	EU219385*
4	*Brighamia rockii*	Nanophanerophyte or Phanerophyte	Hawaiian islands	DQ285257	DQ285140	
5	*Burmeistera crispiloba*	Nanophanerophyte	Neotropics	EF174641	DQ285164	
6	*Burmeistera cyclostigmata*	Nanophanerophyte or Chamaephyte	Neotropics	DQ356147	DQ356213	
7	*Burmeistera domingensis*	Nanophanerophyte or Chamaephyte	Neotropics	DQ356148	DQ356214	
8	*Campanula asperuloides*	Hemicryptophyte	Temp. Eurasia	DQ356117	DQ356170	
9	*Campanula latifolia*	Hemicryptophyte	Temp. Eurasia	EF141027	DQ356169	
10	*Campanula trachelium*	Hemicryptophyte	Temp. Eurasia	DQ356118	DQ356171	
11	*Canarina canariensis*	Geophyte	Temp. Eurasia	DQ356115	DQ356167	
12	*Centropogon cornutus*	Nanophanerophyte or Chamaephyte	Neotropics	DQ356158	DQ356226	
13	*Centropogon dissectus*	Nanophanerophyte or Chamaephyte	Neotropics	EF141026	DQ356215	
14	*Centropogon gamosepalus*	Nanophanerophyte or Chamaephyte	Neotropics	DQ356157	DQ356225	
15	*Centropogon granulosus*	Nanophanerophyte	Neotropics	DQ356152	DQ356220	
16	*Centropogon gutierrezii*	Chamaephyte	Neotropics	AF042658	DQ285165	
17	*Centropogon luteus*	Nanophanerophyte	Neotropics	DQ356151	DQ356219	
18	*Centropogon trichodes*	Nanophanerophyte or Chamaephyte	Neotropics	DQ356149	DQ356217	
19	*Clermontia arborescens*	Nanophanerophyte or Phanerophyte	Hawaiian islands	DQ285258	DQ285141	
20	*Clermontia fauriei*	Nanophanerophyte or Phanerophyte	Hawaiian islands	DQ285259	DQ285142	
21	*Clermontia kakeana*	Nanophanerophyte or Phanerophyte	Hawaiian islands	L18789	DQ356172	EU219386*
22	*Clermontia parviflora*	Nanophanerophyte	Hawaiian islands	DQ285288	DQ285171	
23	*Cyanea acuminata*	Nanophanerophyte	Hawaiian islands	DQ285261	DQ285144	
24	*Cyanea angustifolia*	Nanophanerophyte or Phanerophyte	Hawaiian islands	DQ356119	DQ356173	EU219384*
25	*Cyanea coriacea*	Nanophanerophyte or Phanerophyte	Hawaiian islands	AF042662	DQ285145	
26	*Cyanea floribunda*	Nanophanerophyte	Hawaiian islands	DQ285290	DQ285173	
27	*Cyanea hirtella*	Nanophanerophyte or Phanerophyte	Hawaiian islands	DQ285292	DQ285175	
28	*Cyanea koolauensis*	Nanophanerophyte	Hawaiian islands	DQ356128	DQ356193	
29	*Cyanea kuhihewa*	Nanophanerophyte	Hawaiian islands	DQ285294	DQ285177	
30	*Cyanea leptostegia*	Phanerophyte	Hawaiian islands	DQ285289	DQ285172	
31	*Cyanea pilosa*	Nanophanerophyte	Hawaiian islands	DQ285291	DQ285174	
32	*Delissea rhytidosperma*	Nanophanerophyte	Hawaiian islands	AF042663	DQ285146	
33	*Delissea subcordata*	Nanophanerophyte	Hawaiian islands	DQ285264	DQ285147	
34	*Delissea undulata*	Phanerophyte	Hawaiian islands		DQ356188	EU219389*
35	*Diastatea micranta*	Therophyte	Neotropics	DQ356138	DQ356203	
36	*Downingia bacigalupii*	Therophyte	N America	EF141031	DQ356183	
37	*Downingia insignis*	Therophyte	N America	EF141030	DQ356185	
38	*Grammatotheca bergiana*	Hemicryptophyte	Africa	DQ356116	DQ356168	AF163429
39	*Helianthus annuus - ***Outgroup**	(other)	(other)	AF097517	AJ430967	
40	*Hippobroma longiflora*	Therophyte, Biennal or Hemicryptophyte	Neotropics	DQ356140	DQ356206	
41	*Isotoma axillaris*	Hemicryptophyte	Oceania	DQ268874	DQ285166	
42	*Isotoma fluviatilis*	Hemicryptophyte	Oceania	DQ356161	DQ356230	
43	*Jasione montana*	Biennal or Therophyte	Temp. Eurasia	DQ356120	DQ356174	
44	*Legousia hybrida*	Therophyte	Temp. Eurasia	DQ356163	DQ356234	
45	*Lobelia aguana*	Nanophanerophyte or Chamaephyte	Neotropics	DQ356122	DQ356176	
46	*Lobelia anceps*	Hemicryptophyte		DQ356124	DQ356184	
47	*Lobelia angulata*	Hemicryptophyte	Oceania		AY568754+AY568744	
48	*Lobelia aquatica*	Aquatic	Neotropics	EF141029	DQ356182	
49	*Lobelia arenaria*	Hemicryptophyte	Oceania		AY568756+AY568737	
50	*Lobelia boninensis*	Nanophanerophyte or Herbaceous phanerophyte	Bonin Islands	AF042661	DQ285157	
51	*Lobelia cardinalis*	Hemicryptophyte	N America & Neotropics	AF042659	DQ356231	
52	*Lobelia chinensis*	Hemicryptophyte or Geophyte	Southeast Asia		DQ356228	
53	*Lobelia columnaris*	Nanophanerophyte or Herbaceous phanerophyte	Africa	DQ285275	DQ285158	
54	*Lobelia cordifolia*	Therophyte	Neotropics		DQ356204	
55	*Lobelia coronopifolia*	Chamaephyte	Africa	EF141025	DQ356181	
56	*Lobelia dortmanna*	Aquatic	N America & Temp. Eurasia	DQ356162	DQ356232	EU219388*
57	*Lobelia erinus*	Hemicryptophyte or Therophyte	Africa	L01931	DQ356233	
58	*Lobelia exaltata*	Herbaceous phanerophyte or Hemicryptophyte	Neotropics	DQ356135	DQ356200	EU219383*
59	*Lobelia excelsa*	Nanophanerophyte or Phanerophyte	Neotropics	DQ356146	DQ356212	
60	*Lobelia fistulosa*	Herbaceous phanerophyte or Chamaephyte	Neotropics	DQ356136	DQ356201	EU219387*
61	*Lobelia giberroa*	Phanerophyte	Africa	DQ356127	DQ356192	EU219380*
62	*Lobelia gloria-montis*	Nanophanerophyte	Hawaiian islands	DQ285265	DQ285148	
63	*Lobelia graniticola*	Hemicryptophyte	Africa	DQ356129	DQ356194	
64	*Lobelia gregoriana*	Nanophanerophyte or Herbaceous phanerophyte	Africa		DQ356187	EU219379*
65	*Lobelia hypoleuca*	Nanophanerophyte	Hawaiian islands	DQ356126	DQ356191	
66	*Lobelia irasuensis*	Hemicryptophyte or Geophyte	Neotropics	DQ356121	DQ356175	AY362765
67	*Lobelia kalmii*	Hemicryptophyte	N America	DQ356166	EF126736	
68	*Lobelia kauaensis*	Nanophanerophyte	Hawaiian islands	DQ285267	DQ285150	
69	*Lobelia kraussii*	Hemicryptophyte	Neotropics	EF141024	DQ356179	
70	*Lobelia laxiflora*	Nanophanerophyte or Chamaephyte	N America & Neotropics	DQ356143	DQ356209	AY350631
71	*Lobelia leschenaultiana*	Nanophanerophyte or Chamaephyte	Southeast Asia	DQ356131	DQ356196	
72	*Lobelia macrodon*	Hemicryptophyte	Oceania	EF694730	AY568742	
73	*Lobelia martagon*	Chamaephyte or Hemicryptophyte	Neotropics	DQ356139	DQ356205	
74	*Lobelia nicotianifolia*	Nanophanerophyte or Herbaceous phanerophyte	Southeast Asia	AF042660	DQ285161	
75	*Lobelia niihauensis*	Nanophanerophyte	Hawaiian islands	DQ285268	DQ285151	
76	*Lobelia nummularia*	Hemicryptophyte	Southeast Asia	DQ356164	DQ356235	
77	*Lobelia oligophylla*	Hemicryptophyte	Neotropics	DQ356159	DQ356227	
78	*Lobelia organensis*	Chamaephyte or Hemicryptophyte	Neotropics	DQ285279	DQ285162	
79	*Lobelia perpusilla*	Hemicryptophyte	Neotropics		AY568741	
80	*Lobelia petiolata*	Phanerophyte	Africa	DQ285280	DQ285163	
81	*Lobelia physaloides*	Nanophanerophyte or Chamaephyte	Oceania		AY568757+AY568745	
82	*Lobelia polyphylla*	Nanophanerophyte	Neotropics	DQ356123	DQ356177	AY350633
83	*Lobelia portoricensis*	Chamaephyte or Hemicryptophyte	Neotropics	DQ356142	DQ356208	
84	*Lobelia purpurascens*	Hemicryptophyte or Geophyte	Oceania	DQ356160	DQ356229	
85	*Lobelia rotundifolia*	Nanophanerophyte	Neotropics		DQ356178	
86	*Lobelia roughii*	Hemicryptophyte	Oceania	DQ356165	EF126737	
87	*Lobelia sonderiana*	Hemicryptophyte or Therophyte	Africa	DQ356130	DQ356195	
88	*Lobelia stricklandiae*	Phanerophyte	Africa		DQ356186	EU219381*
89	*Lobelia stricta*	Hemicryptophyte	Neotropics	DQ356141	DQ356207	
90	*Lobelia tomentosa*	Chamaephyte	Africa	EF141028	DQ356180	
91	*Lobelia tupa*	Chamaephyte or Hemicryptophyte	Neotropics	DQ356145	DQ356211	
92	*Lobelia villosa*	Nanophanerophyte	Hawaiian islands	DQ285293	DQ285176	
93	*Lobelia vivaldii*	Nanophanerophyte	Neotropics	DQ268873	DQ285167	
94	*Lobelia xalapensis*	Therophyte	Neotropics	DQ356144	DQ356210	
95	*Lobelia yuccoides*	Nanophanerophyte	Hawaiian islands	DQ356125	DQ356190	
96	*Lysipomia cuspidata*	Chamaephyte	Neotropics	DQ356133	DQ356198	AF054959
97	*Lysipomia sphagnophila*	Chamaephyte	Neotropics	DQ356132	DQ356197	AF054943
98	*Sclerotheca forsteri*	Nanophanerophyte	French Polynesia	DQ285287	DQ285170	
99	*Sclerotheca jayorum*	Nanophanerophyte or Phanerophyte	French Polynesia	DQ285273	DQ285156	
100	*Siphocampylus affinis*	Nanophanerophyte or Chamaephyte	Neotropics	DQ356155	DQ356223	
101	*Siphocampylus brevicalyx*	Nanophanerophyte	Neotropics	DQ356156	DQ356224	
102	*Siphocampylus fulgens*	Nanophanerophyte or Chamaephyte	Neotropics	EF141032	DQ356216	
103	*Siphocampylus giganteus*	Nanophanerophyte or Phanerophyte	Neotropics	DQ356154	DQ356222	
104	*Siphocampylus macropodus*	Nanophanerophyte	Neotropics	DQ356153	DQ356221	
105	*Siphocampylus scandens*	Nanophanerophyte	Neotropics	DQ356150	DQ356218	
106	*Solenopsis laurentia*	Therophyte	Temp. Eurasia	DQ356134	DQ356199	
107	*Trematolobelia kauaiensis*	Nanophanerophyte	Hawaiian islands	DQ285270	DQ285153	
108	*Trematolobelia macrostachys*	Nanophanerophyte or Phanerophyte	Hawaiian islands	DQ356137	DQ356202	EU219382*

Sequences produced in this study are marked with (*); their origin and voucher information are available from their GenBank record.

### Evolution of the giant lobelioids

**Figure 9:**
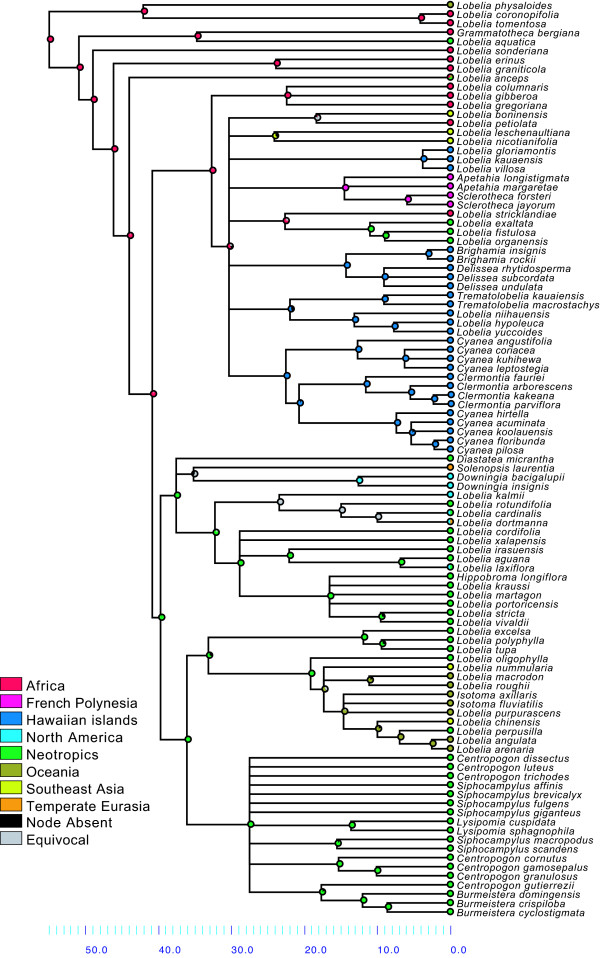
**Ancestral range evolution in relation to time**. Results from ancestral range reconstructions in subfamily Lobelioideae. Methodology as in Figure 7.

The nanophanerophyte habit (Table 2) developed early in the history of the Lobelioideae, most probably several times (at nodes N4, N10 and N12 in Figure 3; with 81.6%, 69.1% and 97.2% of all reconstructions with uniquely best states, respectively), or, less likely, just once (N3; 45.7% of all reconstructions; Figure 7, Table 3). Present day lineages inhabiting the Hawaiian Islands, French Polynesia, southeast Asia and eastern Brazil (N4 in Figure 3) all derive from a single ancestor (Bayesian posterior probability, Bpp = 1.00, Bootstrap support, Bs = 98). Ancestral state reconstructions indicate that this ancestor was most likely confined to Africa and that it was a nanophanerophyte (in 950 and 816 of 1000 reconstructions, respectively; the other reconstructions being ambiguous rather than a different state; see Figures 7 and 9). Indeed, it is in this clade that the truly giant habit occurs, as exhibited by *L. gloria-montis *and *L. rhynchopetalum *(Figure 1). This corroborates previous results, based on fewer species, that giant lobelias are closely related and are ultimately derived from herbaceous ancestors [7,18,21] but contradicts some earlier suggestions the giant habit would be plesiomorphic in the family [6].

Nanophanerophyte ancestors (with buds below 3 m high; see Table 2) have given rise to phanerophytes (buds above 3 m) several times independently. Although several species are strictly phanerophytic (for example, *L. giberroa, L. petiolata, L. stricklandiae, Delissea undulata *and *Cyanea leptostegia*), others often vary in height (for example, *Sclerotheca jayorum, Brighamia insignis, Trematolobelia macrostachys, Cyanea angustifolia *and *Siphocampylus giganteus*). These results indicate that, in the Lobelioideae, height seems to be a more labile state than woodiness.

### Biogeographic history

Geographic range development of the Lobelioideae is depicted in Figure 9. As with life form, distribution is phylogenetically conservative (*P *< 0.001; Figure 8c-d), but even more so given its higher consistency index (0.40) and fewer parsimony steps required for reconstructing the Bayesian cladogram (25).

The MRCA of the Lobelioideae, as well as those of most of the early diverging splits (N1 - N3 in Figure 3), are all reconstructed to Africa with confidence. Range shifts are unambiguously inferred from: Africa to Oceania (*Lobelia physaloides*); Africa to the Neotropics (*Lobelia aquatica*); the Neotropics to Oceania (*Lobelia macrodon - L. arenaria*); and Oceania to southeast Asia (*Lobelia chinensis*). That the Brazilian giant lobelias are together sister to an African species is strongly supported, as previously suggested, by Knox *et al*. [18].

There is no support here for the hypothesis that the giant lobelias arrived in eastern Africa from Asia or the Pacific region [7]. However, the resolution at the base of clade N4 is very poor and the relationships among the strongly supported clades within it - and possibly their biogeographic reconstructions - may substantially change with the addition of more species and sequence data. Indeed, in the analysis by Givnish *et al*. [10], *L. nicotianifolia *(a southeast Asian species) was inferred to be sister to a clade comprising French Polynesian, African and Hawaiian subclades. In the present analysis, that 'basal' position is occupied by African species (*L. columnaris, L. gibberoa *and *L. gregoriana*), although the support for this placement is weak (Bpp = 0.81, Bs < 50). As taxon sampling, sequence regions and phylogenetic methods have all varied considerably between different studies, further investigation is clearly needed in order to reconstruct a solid biogeographic scenario for the large lobelioids.

### Dispersal versus vicariance

Although the exact timing for transcontinental range shifts inferred here are prone to large error margins associated with topological and branch length uncertainties (see the credibility bars at nodes in Figures 4 and 5), all range shifts are estimated to have occurred in the last 50 Ma and, in several cases, presumably much more recently. Given that the final break-up of Gondwanaland took place around ~100 Ma [24], these results provide evidence that long-distance dispersals must have played a major role in shaping the present-day distribution of taxa, a scenario also corroborated by the molecular dating analysis of Givnish *et al*. [10]. This contrasts to early suggestions [6] that distribution patterns may be the result of vicariance caused by continental drift.

**Figure 10:**
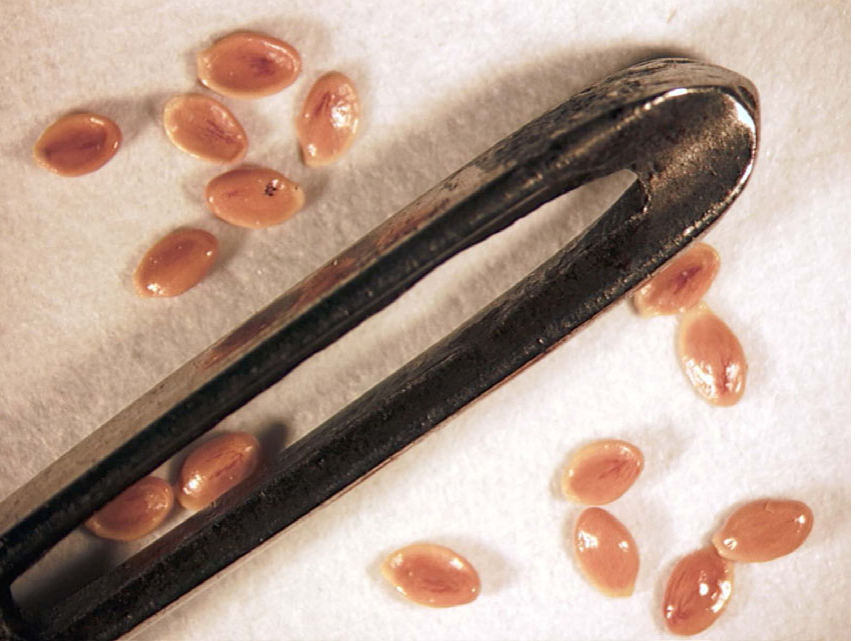
**Tiny seeds crossing long distances**. Lobelioid seeds are extremely small and could presumably be carried over large distances by strong wind currents. The figure shows seeds from the Hawaiian endemic *Clermontia kakeana *around the eye of a needle; one gram contains about 36,000 seeds.

Long-distance dispersal seems plausible for lobelioids, since most species produce large amounts of minuscule seeds. From herbarium specimens, I calculated that 1 g of seed of the Hawaiian endemic *Clermontia kakeana *contains about 36,000 seeds (Figure 10). Although seeds of *Clermontia *and some other Hawaiian genera (*Cyanea *and *Delissea*) are today contained in bird-dispersed berries, other Hawaiian taxa (*Lobelia, Trematolobelia *and *Brighamia*) still possess wind-dispersed capsules, a condition also inferred for the MRCA of the Hawaiian clade by Givnish *et al*. [10]. It is therefore reasonable to conclude that wind dispersal of tiny seeds was the dispersal mode for the ancestor of the Hawaiian lobelioids, as suggested by Givnish *et al*. [10] and that, presumably, the same applies for other lobelioid dispersal events. Millions of tons of dust are transported annually from the Sahara into South America [25], so it is conceivable that, given enough time, lobelioid seeds could also be carried over the 15,000 km separating the Hawaiian Islands and Africa and, subsequently, succeed in germinating and establishing a new population.

### Colonization of the Hawaiian Islands

The earliest diversification of lineages endemic to the Hawaiian Islands corresponds to the crown age of node N6 retrieved in the MrBayes analysis (Figure 3). In the PL analysis, that clade had a mean of 22.4 Ma (16.9 - 29.0 Ma, 95% confidence intervals, CI), whereas in the BEAST analysis the mean was 12.2 Ma (5.74 - 16.2 Ma, 95% Highest Posterior Density, HPD; Table 1). However, in the BEAST analysis, where the Hawaiian taxa had to be constrained as monophyletic (see Methods), the crown group of the whole Hawaiian clade had a mean of 16.4 Ma (9.43 - 23.0 Ma, 95% HPD; see Figure 6b). These ages are only slightly older than the estimates by Givnish *et al*. [10], which varied between 13.6 ± 3.11 Ma and 13.0 ± 1.00 depending on calibration methodology.

The relatively old node ages estimated here may seem surprising, given that the oldest of the modern Hawaiian Islands with well-developed vegetation only dates back to 5.1 Ma [26]. However, they are fully explainable when considering that these islands are part of a much older archipelago formed by the movement of the Pacific plate over a fixed hot spot. A continuous chain of islands have been elevated since 29.8 Ma [26] and lobelioid lineages could have continuously colonized the rising islands and ceased to exist in the subsiding ones. Island hopping by a plant adapted to long-distance dispersal is biologically feasible and has been suggested for the Hawaiian genus *Hillebrandia *in the plant family Begoniaceae [27] and the fern genus *Diellia *[28].

### Correlates of lobelioid diversification

In order to identify possible drivers of biotic diversification, it is essential to infer when and where lineages diversified. Ages and ancestral areas reconstructed here may, therefore, provide a suitable starting point for further examination of particular clades in the Lobelioideae.

One appealing example of how these reconstructions can be useful concerns the SCBL clade (*Siphocampylus, Centropogon, Burmeistera *and *Lysipomia *- clade N12 in Figure 3). This group of mainly nanophanerophytes and chamaephytes comprises over 580 species, which is about half of all Lobelioideae species [21,23,29-31]. The SCBL clade is entirely confined to the Neotropical region, and is particularly rich in species in the Northern Andes. The uplift of the Northern Andes began at ~31 Ma and intensified in the last ~20 Ma, with several discrete phases of uplift which especially affected the Eastern Cordilleras [32]. The temporal match between the Andean uplift and the ages inferred here is striking: onset of diversification was inferred at ~27 Ma (PL) or ~19 Ma (BEAST) (Table 1) and the MRCA of this clade was unambiguously inferred as the Neotropics (Figure 9). Although a correlation in time and space does not necessarily imply causation, there is an emerging consensus that the Andean uplift has played a major role in Neotropical diversification [32-35]. Further studies are clearly needed in order to disentangle the relative role of competing hypotheses of drivers of diversification in this clade, be they mainly abiotic (for example, geotectonic events, climatic fluctuations) or biotic (for example, pollinator interactions, such as those demonstrated for certain species of *Centropogon *and *Burmeistera *[36-40]).

The temporal framework for the Lobelioideae provided here may also contribute to the discussion on the diversification of particular Lobelioideae groups, for which a fine-scale biogeographic analysis has been performed but for which no absolute divergence times have been estimated. One of these is the giant lobelias of eastern Africa, which have been thoroughly studied by Knox and co-workers [7,13,18,41]. Crown ages for the eastern African clade, comprising *Lobelia columnaris, L. gibberoa*, and *L. gregoriana*, estimated here (in PL: mean 22.3 Ma; 13.7 - 30.4 Ma, 95% CI; in BEAST: median 11.2 Ma; 3.09 - 20.8 Ma, 95% HPD) clearly predate the age of most of the mountains in the Eastern and Western African Rifts [7]. These findings influence the interpretation of the tempo and possible processes underlying diversification in the region (see [7] for a detailed account on competing hypotheses and their predictions).

## Conclusion

Let us recapitulate the thought-provoking hypothesis that the giant lobelias of Africa and the Hawaiian Islands converged into the giant life form from herbaceous ancestors in the same way as the giant senecios and the silversword alliance have (pages 84-85 in [4]; Figure 1). The distant relationship between *Dendrosenecio *and the silversword alliance, and their separate diversification, has been generally accepted for more than a century. This is reflected in their taxonomic placement in different tribes based on conspicuous floral dissimilarities and was recently corroborated by molecular phylogenetic analyses [42-45]. In contrast, the results presented here show a very different evolutionary history for the giant form in the subfamily Lobelioideae. The giant lobelioids of Africa and the Hawaiian Islands, together with similarly large species from eastern Brazil, French Polynesia and southeast Asia, are all derived from a single ancestor that was woody and probably African.

Detailed comparative studies are needed in order to evaluate the morphological similarities and differences in habits among and within clades N4, N10 and N12 (Figure 3). A different way of coding for the morphology and habits of species in those clades might affect the reconstruction of the ancestral states for the Lobelioideae. Eventually, more phylogenetically informative sequence data and denser taxon sampling may help elucidate the evolution of this exceedingly diverse plant group.

## Methods

### Choice of taxa and molecular markers

So far, some 2600 sequences classified as belonging to family Campanulaceae have been deposited in GenBank (as of August 2009). In order to assess which of these were correctly placed in subfamily Lobelioideae, I first downloaded all Campanulaceae accessions for the most widely used sequence regions: *rbc*L, *trn*L-F and ITS. Two criteria were then used for choosing the ingroup taxa in order to infer relationships within subfamily Lobelioideae: (i) that the species could be confidently assigned to the Lobelioideae based on the results from the large-scale analyses for the Campanulaceae; and (ii) that the *trn*L-F region was sequenced for that species, since this region has been shown to contain considerably more phylogenetically informative characters than *rbc*L (41.7% as compared to 25.9%, respectively [21]). Based on these criteria, a total of 108 species (including seven outgroup species) were then selected for inferring the phylogeny of the Lobelioideae. This represents an increase of 33 species compared to the high-level phylogeny of the Lobelioideae recently presented by Antonelli [21]. In that analysis, sequences for several taxa exhibiting a large/giant habit had not yet been made available on GenBank. These include representatives from the genera *Apetahia *and *Sclerotheca *(from French Polynesia), *Lobelia boninensis *(from the Bonin Islands), *Lobelia *sect. Galeatella (from the Hawaiian Islands) and *Lobelia nicotianifolia *(from southeast Asia), as well as many other herbaceous species.

In an attempt to increase the phylogenetic resolution among representatives of the giant lobelioids, sequences of the ITS (ITS 1 - 5.8S - ITS 2) of nuclear ribosomal DNA were generated *de novo *for 10 giant species plus *Lobelia dortmanna*. The region was amplified and sequenced with the primers ITS1, ITS2, ITS3, ITS4 [46], and ITS10 [21], following the amplification and sequencing techniques described by Antonelli [21]. Since ITS is one of the fastest evolving molecular markers available today [47-49], its use was expected to add resolution for identifying the relationships between the main groups of giant lobelioids. Table 3 lists all species used in the analyses of subfamily Lobelioideae and indicates the species sequenced for this study.

### Alignment and phylogenetic estimation

Sequences were aligned using the L-INS-I algorithm implemented in the software MAFFT v. 6 [50]. Aligned matrices were inspected manually and all unreliable sequences excluded, iteratively, until all sequences were deemed homologous. The final aligned matrices for Campanulaceae comprised: for *trn*L-F, 452 sequences and 1541 characters; for ITS, 445 sequences and 1197 characters; and for *rbc*L, 438 sequences and 1401 characters.

Maximum Likelihood trees were inferred in the software Garli 0.960 [51] by performing 10 independent runs with the default settings. The analyses were performed in the CIPRES cluster at the San Diego Supercomputer Center http://www.phylo.org/portal2 and the results visualized using the Interactive Tree of Life facility http://itol.embl.de. MrModeltest [52,53] was used to select the best-fitting evolutionary model for each sequence region. The Partition Homogeneity Test [54] was applied to test for conflicting phylogenetic signal among these regions by performing a heuristic search with 5000 replicates, 100 random addition sequences, TBR branch swapping and saving up to 50 trees per replicate in PAUP* version 4.0b10 [55]. Since the test did not approach significance (*P *= 0.962), the three markers were combined for the subsequent analyses. A bootstrap analysis was run in PAUP under the maximum parsimony criterion, by sampling 10000 replicates, with 100 random addition sequences and saving one tree per replicate. A Bayesian phylogenetic analysis was then conducted with the software MrBayes v. 3.1 [56], performing two parallel runs of 20 million generations each, using four chains, sampling every 1000 generations and saving branch lengths. The performance of the analyses was evaluated using the software Tracer v.1.4.1 [57] and node frequency statistics calculated on 20000 post burn-in trees.

### Molecular dating

Several methods are currently available for estimating divergence times in a phylogeny [58,59]. In order to compare the results from two widely used methods, divergence times were estimated using the Bayesian relaxed clock and PL approaches [60,61]. Both methods have the advantage of enabling direct calibration on one or more nodes of a phylogeny. A major difference between them, however, is that PL assumes that rates are auto-correlated (inheritable), whereas in Bayesian relaxed clock dating each branch is allowed to evolve at its own rate.

PL estimations were done using the software r8s [61,62]. An automated cross-validation algorithm was run to identify the optimal smoothing value for the final analysis, with log_10 _increments of 0.1 and using the Truncated Newton method implemented in r8s. For estimating the effects of phylogenetic uncertainty on node age estimations, 1000 trees from the stationary sample of Bayesian trees were independently dated and their statistics (mean and 95% confidence interval values) computed for each node of the Bayesian consensus tree using the software TreeAnnotator [63].

For the Bayesian dating analysis, five runs of 10 million generations each were performed in the software BEAST v.1.4.8 [22] at the Computational Biology Service Unit hosted by Cornell University, USA http://cbsuapps.tc.cornell.edu. The analysis assumed a pure birth (Yule) process, since this tree prior is most suitable for inferring relationships between individuals from different species [22]. The performance of the analysis (convergence of the independent runs, effective sample sizes) was evaluated using Tracer v.1.4.1 [22]. Mean and 95% HPD intervals of ages were then calculated from 40,000 post burn-in trees using the software TreeAnnotator v.1.4.8 [22], and visualized using FigTree v1.2.2 [63].

Three simultaneous calibration points were applied in both analyses. (i) Since the fossil record of Campanulaceae is exceptionally scarce [64], the root of the Asterales tree (see [65]) was calibrated at 93 Ma, as estimated under a major study including 83 asterid families and based on six plastid markers and six fossil calibrations [66]. Reliability of this age was recently corroborated by a study that employed the same set of fossils, but a different set of sequence regions and taxa (mean 94 Ma; [67]). In the PL analysis, 93 Ma was set as a fixed calibration, whereas in the BEAST analyses it constituted a normally distributed prior with mean 93 Ma and standard deviation of 1.0. (ii) An unchallenged fossil *Campanula *from the Miocene of Poland [68] was used to impose a minimal age constraint of 5.33 Ma for subfamily Campanuloideae. (iii) A 29.8 Ma maximum age was imposed for the radiation of the endemic Hawaiian taxa. This corresponds to the age of the oldest island of the Hawaiian Ridge (Kure), after which a continuous chain of islands has been available for propagules of the highland Hawaiian biota. In the PL analysis, this provided minimal ages for the five strongly supported (Bpp = 1.00) crown groups of Hawaiian species identified in the MrBayes analysis (see Figure 4, Results). In order to be able to use the latter as a calibration point in the BEAST analyses, it first was necessary to constrain all Hawaiian species to be monophyletic (failure to do so caused the program to crash, apparently due to a failure in generating starting trees). Since the monophyly of the Hawaiian lobelioids was demonstrably strongly supported [Bpp = 1.00, 8, 10], this *a priori *constraint was not expected to significantly influence the results.

### Biogeographic and character evolution analyses

One problematic aspect in referring to 'giant lobelioids' is how we should characterize these plants morphologically. Giant lobelias have been described as:

'pachycaul plants; often long-lived and pliestesial; of large stature and bulk; with dense apical rosettes of typically sessile leaves, which may close every night and are retained even after they die for insulation and fire protection; with nightly closing, large racemose inflorescences; with a large apical bud of developing leaves to protect young tissue; which secrete ice-nucleating polysaccharide fluids that prevent frost damage; and a hollow pith for internal water storage that narrows basipetally'. (see [7] and references therein [23,69,70]).

**Figure 11:**
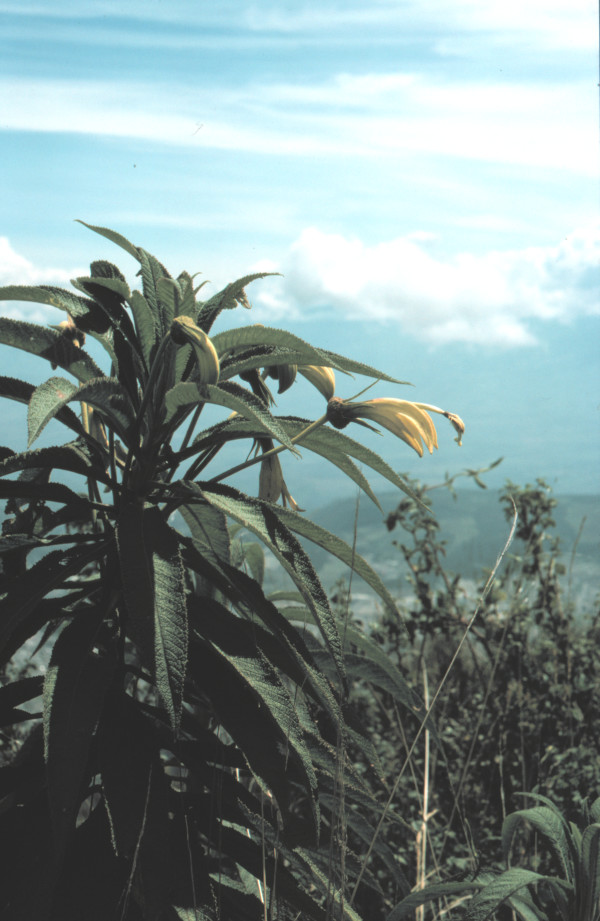
**Large habit attained elsewhere**. Although truly giant lobelioids (with a thick stem and a large terminal leaf rosette) all belong to the same clade, some Neotropical species in the genera *Siphocampylus, Centropogon *and *Burmeistera *can be rather tall shrubs. The Andean *Siphocampylus giganteus *portrayed here is one example which has been coded as varying between nanophanerophyte and phanerophyte. [Credit: Lennart Andersson].

These features correctly describe several of the eastern African species but they do not apply to all of them, let alone to other large lobelioids elsewhere (for example, the Andean species *Siphocampylus giganteus*, Figure 11). To overcome this problem, life form was coded here according to the recent checklist of the Campanulaceae by Lammers [23] (Table 2). Besides being a comprehensive and up-to-date account on life form and distribution for all species, this work provides a consistent framework for evaluating evolutionary traits in the family. The current distribution of all species was compiled from the same source [23], but simplified into eight larger operational units: Africa, French Polynesia, the Hawaiian Islands, temperate North America, the Neotropical region including temperate South America, Oceania, southeast Asia and temperate Eurasia. For both characters, the outgroup taxon (*Helianthus*) was coded as having a different state to any of the ingroup taxa, to prevent biasing the reconstruction of basal nodes on the tree (since *Helianthus *is used here to root the Campanulaceae tree but it is certainly not the sister species to the Campanulaceae). Life form and distribution for all species analysed are listed in Table 3.

The most widely used methods of ancestral area reconstruction that are able to take into account phylogenetic uncertainty and multistate characters are Fitch Parsimony (FP), implemented in the software Mesquite v. 2.7 [71] and Bayes-DIVA [72,73]. These two methods are based on very different biogeographic evolutionary models. FP constrains ancestors to be monomorphic (that is, restricted to single areas) and models changes in distribution from ancestor to descendant as a change in character state, equivalent to dispersal between single areas. It thus implements a dispersalist explanation. In contrast, DIVA allows widespread distributions at ancestral nodes. Although the maximum number of areas can be constrained in DIVA, single-area ancestors are not allowed and widespread distributions are always divided at speciation events by vicariance. Thus DIVA favours a more vicariant explanation [74]. Based on the molecular dating results obtained (see Results and Discussion), and taking into consideration that the Hawaiian archipelago has never been connected to any land mass [26], it is clear that long-distance dispersals have played a crucial role in shaping the distribution of the Lobelioideae. This indicated that Fitch optimization was a more suitable method for inferring ancestral ranges of lobelioid nodes than DIVA. Mesquite v. 2.7 [71] was therefore used for reconstructing both ancestral areas and ancestral life forms. To take into account phylogenetic uncertainty, reconstructions were performed on 1000 trees from the stationary Bayesian tree sample, using the Maximum Parsimony criterion and counting all trees with uniquely best states. The results were summarized by computing the relative frequencies of ancestral area reconstructions for each node of the Bayesian consensus cladogram of the Lobelioideae.

### Tests of phylogenetic conservatism

In order to test whether life form and geographic distribution were phylogenetically conservative, I first calculated the number of parsimony steps necessary to explain the occurrence of each character on a particular tree, repeating this for 1000 trees randomly chosen from the post burn-in Bayesian sample (the same sample used for the dating and character state reconstruction analyses). I then generated 1000 trees with the same topology as the Bayesian 50% majority-rule consensus but with character states randomly shuffled among the tips of each tree, while keeping their relative frequency constant. The values obtained after reconstructing the characters on the simulated data set were used to compute 99% credibility intervals, to which the observed numbers of parsimony steps for each respective character could be compared. The analyses were performed in Mesquite v. 2.7 [71].

## Abbreviations

BEAST: Bayesian evolutionary analysis by sampling trees; Bpp: Bayesian posterior probability; Bs: Bootstrap support; CI: Confidence interval; HPD: Highest Posterior Density; ITS: internal transcribed spacer; Ma: Mega-annum (million years); MRCA: most recent common ancestor; PL: Penalized Likelihood; SCBL: *Siphocampylus, Centropogon, Burmeistera, Lysipomia*.
